# Synthesis of CrO_*x*_/C catalysts for low temperature NH_3_-SCR with enhanced regeneration ability in the presence of SO_2_†

**DOI:** 10.1039/c7ra09680a

**Published:** 2018-01-22

**Authors:** Shuohan Yu, Sheng Xu, Bowen Sun, Yiyang Lu, Lulu Li, Weixin Zou, Peng Wang, Fei Gao, Changjin Tang, Lin Dong

**Affiliations:** Key Laboratory of Mesoscopic Chemistry of MOE, School of Chemistry and Chemical Engineering, Nanjing University Nanjing 210093 PR China donglin@nju.edu.cn gaofei@nju.edu.cn; Jiangsu Key Laboratory of Vehicle Emissions Control, Center of Modern Analysis, Nanjing University Nanjing 210093 PR China; National Laboratory of Solid State Microstructures, College of Engineering and Applied Sciences, Collaborative Innovation Center of Advanced Microstructures, Nanjing University Nanjing 210093 China

## Abstract

Chromium oxide nano-particles with an average diameter of 3 nm covered by amorphous carbon (CrO_*x*_/C) were successfully synthesized. The synthesized CrO_*x*_/C materials were used for the selective catalytic reduction of NO_*x*_ by NH_3_ (NH_3_-SCR), which shows superb NH_3_-SCR activity and in particular, satisfactory regeneration ability in the presence of SO_2_ compared with Mn-based catalysts. The as-prepared catalysts were characterized by XRD, HRTEM, Raman, FTIR, BET, TPD, TPR, XPS and *in situ* FTIR techniques. The results indicated presence of certain amounts of unstable lattice oxygen exposed on the surface of CrO_*x*_ nano-particles with an average size of 3 nm in the CrO_*x*_/C samples, which led to NO being conveniently oxidized to NO_2_. The formed NO_2_ participated in NH_3_-SCR activity, reacting with catalysts *via* a “fast NH_3_-SCR” pathway, which enhanced th NH_3_-SCR performance of the CrO_*x*_/C catalysts. Furthermore, the stable lattice of the CrO_*x*_ species made the catalyst immune to the sulfation process, which was inferred to be the cause of its superior regeneration ability in the presence of SO_2_. This study provides a simple way to synthesize stable CrO_*x*_ nano-particles with active oxygen, and sheds light on designing NH_3_-SCR catalysts with highly efficient low temperature activity, SO_2_ tolerance, and regeneration ability.

## Abstract 2

Novel CrO_*x*_@C catalyst with both remarkable NH_3_-SCR activity and satisfactory regeneration ability in the presence of SO_2_.

## Graphical abstract

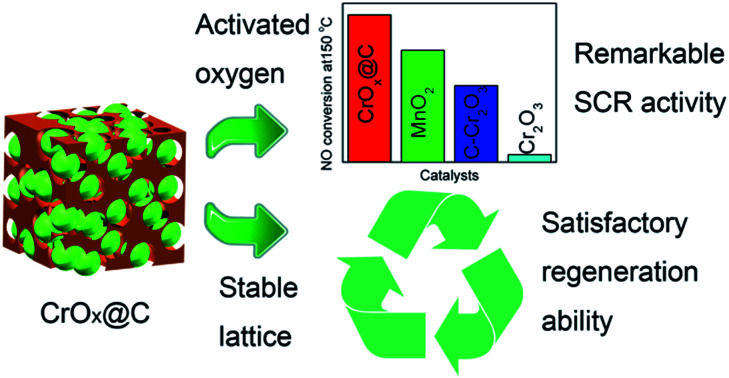

## 1. Introduction

Low-temperature NH_3_-SCR (<200 °C), which can be located downstream of electrostatic precipitators and even desulfurizers, where most of SO_2_ and dust have been removed, has been paid increasing attention in the past few decades.^1–4^ Various transition metal oxides exhibit satisfactory activity for low-temperature NH_3_-SCR, such as Mn, Fe, Cu, and Co.^2,5–9^ Among them, Mn-based catalysts have become a focus for their excellent low-temperature activity and inherent environment-friendly nature.^4,10–12^ According to literature, Mn-based catalysts have a unique advantage for low-temperature SCR (<200 °C) in contrast with other competitors. For example, Hu *et al.* reported a Co–Mn/TiO_2_ catalyst with superior NH_3_-SCR activity at 200 °C.^11^ Mn–Ce–Ti mix-oxide catalysts were recorded to exhibit an operating temperature window from 150 °C to 200 °C.^12^ We also reported Mn–Fe–Ti mix-oxide catalysts with satisfactory NH_3_-SCR activity from 100 °C to 350 °C.^5^ However, the poor SO_2_ resistance performance of Mn-based catalysts limits their practical application.

According to the previous reports,^4,13–15^ the tolerance of metal oxide based catalysts to SO_2_ depends on the type and oxidation state of the deposited metal, the nature of the support, and the type of reducing agent. In general, the rapid deactivation of NH_3_-SCR catalysts involves two main mechanisms. One is the formation of ammonium salts.^14,16–22^ The SO_2_ in the feed gas can be oxidized to SO_3_ on the surface of the catalysts, and the formed SO_3_ would respond to NH_3_ and water in feed gas transforming to NH_4_HSO_4_. The formed NH_4_HSO_4_ would deposit on the surface of the catalysts, cover active sites, block pores of the catalysts, and result in the deactivation of the catalysts. The other fact is the irreversible sulfation of the active phase.^14,16,18–23^ For most transition metal oxides usually reported as NH_3_-SCR catalysts, such as Mn, Fe, Cu, and Co, all sulfating processes are spontaneous according to their Gibbs free energy values (Table S1†). In the sulfation process, formation of a metal sulfate requires breaking the metal oxide lattice. It is reasonable to predict that the more stable the metal oxides, the more difficult it is to break the metal oxide lattice and thus, harder is the sulfation of metal oxide. In general, the metal oxide with a high melting point has a stable crystal lattice. Thus, the melting point can be an indicator of the stability of the crystal lattice of metal oxides.^24^ In the case of Mn-based catalysts, MnO_2_ has a low melting point (Table S1†), indicating its unstable structure, which is the cause of the severe irreversible sulfation of MnO_2_ in the NH_3_-SCR process, particularly in the low temperature range (<200 °C).^13,14,25–28^ Cr_2_O_3_, which has the highest melting point among the transition metal oxides with NH_3_-SCR activity (listed in Table S1†), was expected to have resistance to the sulfation process. Although the low NH_3_-SCR activity of crystalline Cr_2_O_3_ is unsatisfactory, amorphous Cr_2_O_3_ exhibits superb low temperature NH_3_-SCR activity, according to literature.^29–33^ Thus, it appears to be a promising strategy to design a SCR catalyst with both low temperature activity and SO_2_ tolerance *via* enhancing the catalytic activity of crystalline Cr_2_O_3_.

Compared with traditional metal oxide catalysts with a large particle size, nano-sized catalysts often have significant amounts of unsaturated-coordinated atoms exposed on their surfaces. Unsaturated-coordinated atoms are usually active; thus, nano-sized catalysts exhibit unique redox ability and enhanced catalytic activity.^34–36^ Therefore, decreasing the size of catalysts appears to be a promising strategy to enhance low temperature NH_3_-SCR performance of crystalline Cr_2_O_3_. However, nano-particle materials have the disadvantage of instability and tend to aggregate due to their high surface energy and their abundant surface unsaturated atoms. Recently, metal oxide nano-particles catalysts derived from MOFs material were reported.^37–39^ Through a thermal decomposition process under controlled atmosphere, MOFs built from metal ions or nodes and polyfunctional organic ligands can transform into nano-materials, including nano-particles, single atoms, and metal oxide clusters. Wu *et al.*^40^ synthesized Co nano-particles and single atoms from Co MOF and Co/Zn bimetallic MOF. Similar results were also reported by Li *et al.*^37^ and Sun's group.^41^ The carbon from the organic ligands of MOFs remains in materials and can protect metal oxide nano-particles from aggregation.

Herein, novel CrO_*x*_ nano-particles covered by amorphous carbon (CrO_*x*_/C) have been synthesized by a MOFs assisted process for low temperature NH_3_-SCR. MIL-101, with a metal node of 3 Cr atoms,^42,43^ was employed as a precursor. The results of the catalytic tests for NH_3_-SCR showed that the prepared CrO_*x*_ catalyst exhibited satisfactory activity and superior regeneration ability. According to a series of characterizations, the CrO_*x*_/C catalyst was observed to be composed of CrO_*x*_ nano-particles with an eskolaite phase and activated lattice oxygen. It was deduced that the activated lattice oxygen was closely related to the enhanced NH_3_-SCR activity of the CrO_*x*_/C catalyst. The stable lattice of the eskolaite phase-CrO_*x*_ inhibited the sulfating process, thus causing the SO_2_ tolerance and regeneration ability. To the best of our knowledge, it is the first time that a non-Mn catalyst with excellent low temperature NH_3_-SCR activity and remarkable regeneration ability has been reported. This study provides a simple route to synthesize stable CrO_*x*_ nano-particles with active oxygen and shed light on designing low temperature NH_3_-SCR catalysts with SO_2_ tolerance and regeneration ability.

## 2. Experimental details

### 2.1. Preparation of catalysts

Typically, MIL-101(Cr) was prepared by reacting terephthalic acid (332 mg, 2.0 mmol) with Cr(NO_3_)_3_·9H_2_O (800 mg, 2.0 mmol) and de-ionized water (9.5 mL) at 220 °C for 8 h. Microcrystalline green powders of MIL-101(Cr) were produced during the reaction. The obtained powders were washed by ammonium hydroxide, water, and ethanol, in sequence, 3 times each. The powders were dried and calcined at a certain temperature for 4 h under N_2_ flow, and the heating rate was set at 1 °C min^−1^. Finally, the cooled sample was exposed to air and denoted as Cr_2_O_3_/C-*X*, in which *X* represents the calcining temperature.

As reported in ref. 44, a Cr_2_O_3_ sample was obtained by calcining Cr(NO_3_)_3_·9H_2_O at 450 °C for 4 h. MnO_2_ was purchased from Aladdin and was used without further purification. An active carbon supported Cr_2_O_3_ catalyst was synthesized through a wetness impregnation process. Active carbon (1.00 g) was dispersed into de-ionized water (50 mL) containing Cr(NO_3_)_3_·9H_2_O (9.92 g). The turbid solution was oil-bath heated at 110 °C until the water was totally evaporated. The dried powders were calcined at 450 °C for 4 h under a N_2_ flow and the obtained sample was noted as Cr_2_O_3_/C-WI.

### 2.2. Characterizations

The X-ray diffraction (XRD) patterns of the catalysts were studied using an XRD-6000 X-ray diffractometer (Shimadzu). X-ray fluorescence (XRF) analysis was performed on an ARL-900 X-ray fluorescence analyzer. FTIR analysis was carried out using a NEXUS870 spectrometer (NICOLET, America). Raman spectra were measured at a resolution of <1 cm^−1^ using a JY Labram HR 800 spectrophotometer equipped with an argon-ion laser source and an air-cooled CCD detector. N_2_ adsorption/desorption isotherms of the catalysts were obtained at −196 °C using an ASAP2020 physical adsorption instrument (Micromeritics) to calculate the BET surface area of the catalysts. TEM analysis was performed on a double-aberration corrected Titan™ cubed G2 60-300 S/TEM equipped with Super-X™ technology. X-ray energy dispersive spectroscopy (EDS) mappings were acquired using the Super-X EDS system, which is composed of four silicon drift detectors covering 0.7 s rad collection.

NH_3_-temperature programmed desorption (NH_3_-TPD) experiments were performed using a multifunction chemisorption analyzer, equipped with a thermal conductivity detector (TCD). Samples were pretreated under a NH_3_–N_2_ flow (NH_3_ 1%) at 150 °C for 1 h and were heated under N_2_ flow; the heating rate was set at 10 °C min^−1^.

O_2_-temperature programmed desorption (O_2_-TPD) experiments were performed using a multifunction chemisorption analyzer, equipped with a thermal conductivity detector (TCD). Samples were pretreated under O_2_–He flow (O_2_ 25%) at 25 °C for 1 h and were heated under a He flow; the heating rate was set at 10 °C min^−1^.

H_2_-temperature programmed reduction (H_2_-TPR) of the catalysts was recorded using a chemisorption analyzer. Samples were pretreated under a N_2_ flow at 200 °C for 1 h, and were heated under a H_2_–Ar flow (H_2_, 7%); the heating rate was set at 10 °C min^−1^.

X-ray photoelectron spectroscopy (XPS) measurements were performed using a PHI 5000 VersaProbe spectrophotometer. The contents of the metal ions were measured *via* an inductive coupled plasma emission spectrometer (Optima 5300DV, PE). Energy referencing was accomplished by setting the adventitious carbon peak to 284.6 eV. The *ex situ* XPS details are described below. The sample was treated under a certain atmosphere for a certain time in a reaction chamber connected with the intro chamber of the XPS instrument. Following this, the reaction chamber was vacuumized and the treated sample was transferred to the XPS instrument without exposure to air.

The *in situ* DRIFT experiments were performed on a Nicolet Nexus 5700 FTIR spectrometer using a diffuse reflectance attachment (HARRICK) equipped with a reaction cell (ZnSe windows). The number of scans was 32 at a resolution of 4 cm^−1^ and the spectra were presented as Kubelka–Munk function, referred to the background spectra of the recorded catalyst in N_2_.

### 2.3. NO oxidation tests

The NO oxidation tests were performed in a fixed-bed reactor with 0.2 g catalyst. The feed gas contained 500 ppm NO and 5 vol% O_2_ with N_2_ as the balance gas. The total flow rate of the feed gas was 100 mL min^−1^, corresponding to a space velocity of approximately 30 000 h^−1^. Including NO and NO_2_, the effluent gases were continuously analyzed at 150 °C by an online Thermofisher IS10 FTIR spectrometer equipped with a 2 m path-length gas cell (250 mL volume).

### 2.4. NH_3_-SCR activity, SO_2_ poisoning, and regeneration tests

The NH_3_-SCR activity tests were performed in a fixed-bed reactor with 0.2 g catalyst. The feed gas contained 500 ppm NO, 500 ppm NH_3_, 5 vol% O_2_, 50 ppm SO_2_ (when used), 5 vol% H_2_O (when used) and N_2_ as the balance gas. The total flow rate of the feed gas was 100 mL min^−1^, corresponding to a space velocity of approximately 30 000 h^−1^. SO_2_ poisoning and regeneration tests of catalysts were carried out at 150 °C. Including NO, NH_3_, NO_2_, and N_2_O, the effluent gases were continuously analyzed at 150 °C using an online Thermofisher IS10 FTIR spectrometer equipped with a 2 m path-length gas cell (250 mL volume).

### 2.5. Regeneration of SO_2_ poisoned catalysts

SO_2_ poisoned catalysts were regenerated at 300 °C for 30 min, and then were cooled down to room temperature. All the heat treatments were carried out under N_2_ atmosphere.

The sulfating of metal oxide catalysts during SO_2_ poisoning was investigated by inductively coupled plasma-emission spectroscopy (ICP).

The SO_2_-poisoned catalyst (0.2 g) was washed with deionized water for 5 times. The eluate was collected and diluted to 50 mL. The diluted eluate was investigated by ICP analysis. The contents of metal sulfate *m*_MSO_4__ were calculated using the equation below.
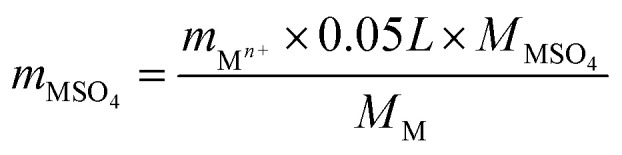
where, *m*_M^*n*+^_ is the content of the metal ion, *M*_MSO_4__ was obtained *via* ICP analysis and *M*_M_ is the molar mass of metal sulfate, which also corresponds to the molar mass of the metal ion.

For Cr oxide, *m*_Cr_2_(SO_4_)_3__ = *m*_Cr^3+^_ × 0.188

For Mn oxide, *m*_MnSO_4__ = *m*_Mn^2+^_ × 0.137

## 3. Results and discussion

### 3.1. NH_3_-SCR performance, SO_2_ tolerance, and regeneration

**Fig. 1:**
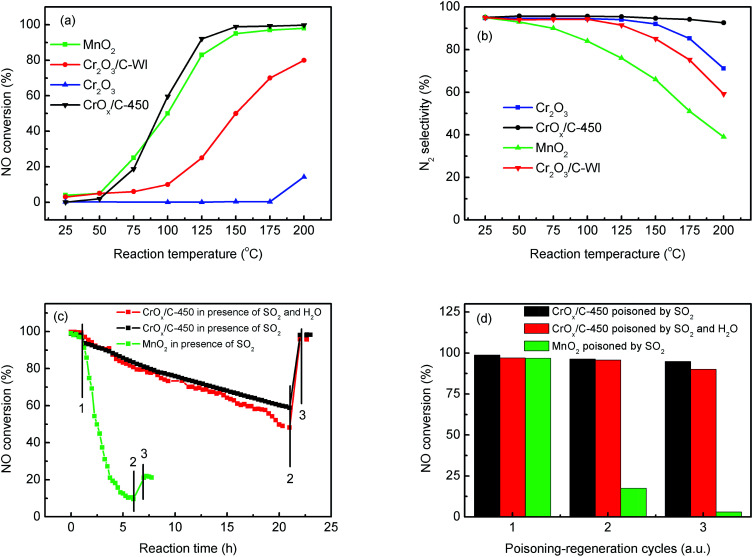
NH_3_-SCR activity of catalysts: (a) NO conversion, (b) N_2_ selectivity, (c) SO_2_ poisoning test (step 1, 2, and 3 present SO_2_ and H_2_O (when used) on, SO_2_ and H_2_O (when used) off, and after regeneration), and (d) regeneration ability.

**Fig. 2:**
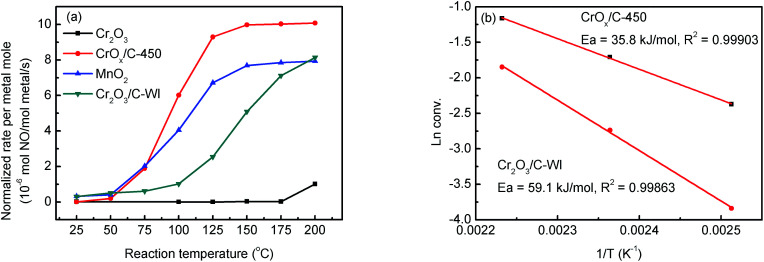
(a) Normalized rates per metal mole of catalysts, (b) apparent active energy of CrO_*x*_/C-450 and Cr_2_O_3_/C-WI catalysts.

The NH_3_-SCR activities of the CrO_*x*_ samples derived from various precursors and MnO_2_ are shown in Fig. 1 and S1†. As it was anticipated, all the catalysts derived from MIL-101 exhibited superior activities than pure Cr_2_O_3_. Remarkably, CrO_*x*_/C-450, parallel to MnO_2_, exhibited a wide operation temperature window from 125 °C to 200 °C. The activity of Cr_2_O_3_/C-WI was enhanced compared with pure Cr_2_O_3_, while it was much lower than CrO_*x*_/C-450. This extraordinarily low temperature NH_3_-SCR performance makes CrO_*x*_/C-450 the best catalyst among all the samples derived from MIL-101. To evaluate the NH_3_-SCR performances on the catalysts more precisely, the normalized rates per mole transition metal were calculated and the results are displayed in Fig. 2a. The catalytic activity order is CrO_*x*_/C-450 > Mn_2_O_3_ > Cr_2_O_3_/C-WI > Cr_2_O_3_. In addition, the apparent active energies of CrO_*x*_/C-450 and Cr_2_O_3_ based catalysts were obtained when NO conversions were limited to low conversion (Fig. 2b and Table S2†). The apparent active energy of NH_3_-SCR on the CrO_*x*_/C-450 catalyst was lower than those of SCR on Cr_2_O_3_/C-WI and Cr_2_O_3_, which further confirms the superb catalytic activity of the CrO_*x*_/C-450 sample. Based on the kinetics data listed in Tables S3 and S4,† apparent kinetics equations of NH_3_-SCR on CrO_*x*_/C-450 and Cr_2_O_3_/C-WI catalysts were obtained (Fig. S2†). For CrO_*x*_/C-450, *r* = [NH_3_]^0.586^[NO]^0.964^ at 150 °C, while for Cr_2_O_3_/C-WI, *r* = [NH_3_]^0.433^[NO]^0.092^. The different reaction rate equations of NH_3_-SCR on CrO_*x*_/C-450 and Cr_2_O_3_/C-WI catalysts indicated their different reaction mechanisms, which may result in different NH_3_-SCR performance of CrO_*x*_/C-450 and Cr_2_O_3_/C-WI catalysts. The N_2_ selectivity of CrO_*x*_/C-450 remained at a high level (over 90%) in its operation temperature window, while those of Cr_2_O_3_ and MnO_2_ were very poor. This indicated that side reactions such as the formation of N_2_O hardly occurred on the CrO_*x*_/C-450 catalyst. SO_2_- and H_2_O-tolerance of CrO_*x*_/C-450 was further tested and the results are presented in Fig. 1c and S1c.† CrO_*x*_/C-450 exhibited over 80% NO conversion within 24 h in the presence of H_2_O, indicating its satisfactory water tolerance. When SO_2_ was introduced into the feed gas, the activity of CrO_*x*_/C-450 gradually dropped to 60% within 20 h, while it dropped to 50% in 20 h when SO_2_ and H_2_O co-existed in the feed gas. The activity of CrO_*x*_/C-450 could be recovered after a heat treatment at a temperature as low as 300 °C. This indicated that the poisoning effect of SO_2_ on CrO_*x*_/C-450 could be accelerated by H_2_O, while H_2_O hardly influenced the regeneration ability of CrO_*x*_/C-450. In contrast, the MnO_2_ catalyst deactivated rapidly upon SO_2_ introduction in 6 h, and this process was irreversible. It was demonstrated that the CrO_*x*_/C-450 catalyst has satisfactory SO_2_-tolerance. The regeneration ability of CrO_*x*_/C-450 was further studied. As shown in Fig. 1d, over 90% of the catalytic activity of CrO_*x*_/C-450 could be recovered as compared to that of the fresh catalyst after 3 poisoning–regeneration cycles irrespective of whether H_2_O was introduced by regeneration at 300 °C in flowing N_2_, while that of MnO_2_ catalyst dropped dramatically through only 1 poisoning–regeneration cycle, which indicated the remarkable regeneration ability of the CrO_*x*_/C-450 sample.

### 3.2. Structural information

To investigate the NH_3_-SCR on the catalysts, the structural information of catalysts was necessary. XRD, XRF, FTIR, Raman, and TEM analyses were carried out to investigate the structural properties of the catalysts synthesized from MIL-101(Cr). The XRD patterns of all samples are shown in Fig. S3.† In the XRD pattern of the precursor, sharp and distinct peaks attributed to the MIL-101 phase were detected, which is in agreement with the data reported by Jhung *et al.*^43^ When MIL-101 was heated in N_2_-flow, the XRD peaks of MIL-101 became weak and gradually disappeared with an increase in temperature. When the calcining temperature reached 450 °C, the peaks of the MIL-101 phase disappeared completely and replaced with wide and weak peaks at 24.5°, 33.5°, and 36.1°, which belonged to the (012), (104), and (110) plane, respectively, of the eskolaite phase (PDF#38-1479) as well as Cr_2_O_3_/C-WI.^45^ This indicated that the MIL-101 structure could be destroyed through the calcining process, accompanied with the formation of the eskolaite phase. Furthermore, the CrO_*x*_/C-450 sample was found to have a small particle size based on its broad XRD peaks.

FTIR and Raman analyses were carried out to study the carbon species in the catalysts. As shown in Fig. S4a,† the bands at 3300 cm^−1^, 1600 cm^−1^, and 1400 cm^−1^ were identified in the FTIR spectra of MIL-101. The broad band at 3300 cm^−1^ arises due to the stretching vibration of the surface –OH groups. The two bands at 1600 and 1400 cm^−1^ could be attributed to the stretching vibration of the –COO group of the organic linkers of MIL-101. However, the intensity of bands at 1600 cm^−1^ and 1400 cm^−1^ decreased on increasing the calcination temperature. This indicates that the organic linker begins to decompose and carbonize when the calcining temperature increases. The carbonization process was further investigated by Raman analysis (Fig. S4b†). Wide bands at 1360 and 1590 cm^−1^, corresponding to the D-band and G-band of MIL-101, were detected in the samples calcined at low temperatures, such as 350 and 400 °C. When the calcining temperature increased, the G-band gradually disappeared and the D-band still remained, which indicated the loss of the ordered structure of MOFs and the formation of amorphous carbon. In the spectrum of Cr-550, the G-band disappeared absolutely and only a wide D-band was observed. This illustrates that the organic linker carbonized and transformed to amorphous carbon during the calcining process, which accompanied with the destruction of the MOFs structure.

The elemental contents of catalysts were studied *via* XRF analysis. Since the organic species in MIL-101 completely transformed to amorphous carbon in CrO_*x*_/C-450, as mentioned before, the ignition loss in XRF analysis of CrO_*x*_/C-450 was believed to be the result of carbon species burning. Thus, the elemental contents of the catalysts could be calculated from the XPF data and the results are shown in Table 1. The CrO_*x*_/C-450 catalyst consisted of 19.2% Cr, 28.7% O, and 52.0% C. As it was designed, the elemental contents of Cr_2_O_3_/C-WI were similar to those of CrO_*x*_/C-450.

**Table 1:** Element contents and surface areas of catalysts

Sample	Contents^a^ (wt%)		Elements contents (at%)			Surface area^b^ (m^2^ g^−1^ catalyst)		
	Cr_2_O_3_	Ignition loss	Cr	O	C	Sample	Carbon support^c^	Active material
CrO_*x*_/C-450	70.2	29.8	19.2	28.7	52.0	256	784	20.8
Cr_2_O_3_/C-WI	69.5	30.5	18.9	28.4	52.7	279	897	9.9
Cr_2_O_3_	99.9	0.1	40.0	60.0	—	28	—	—

a Contents of catalysts were obtained from XRF analyses.

b Surface areas of catalysts were obtained from N_2_ adsorption/desorption analyses.

c Carbon support of CrO_*x*_/C-450 catalyst was obtained by washing CrO_*x*_/C-450 using hydrochloric acid. Carbon support of Cr_2_O_3_/C-WI is active carbon.

**Fig. 3:**
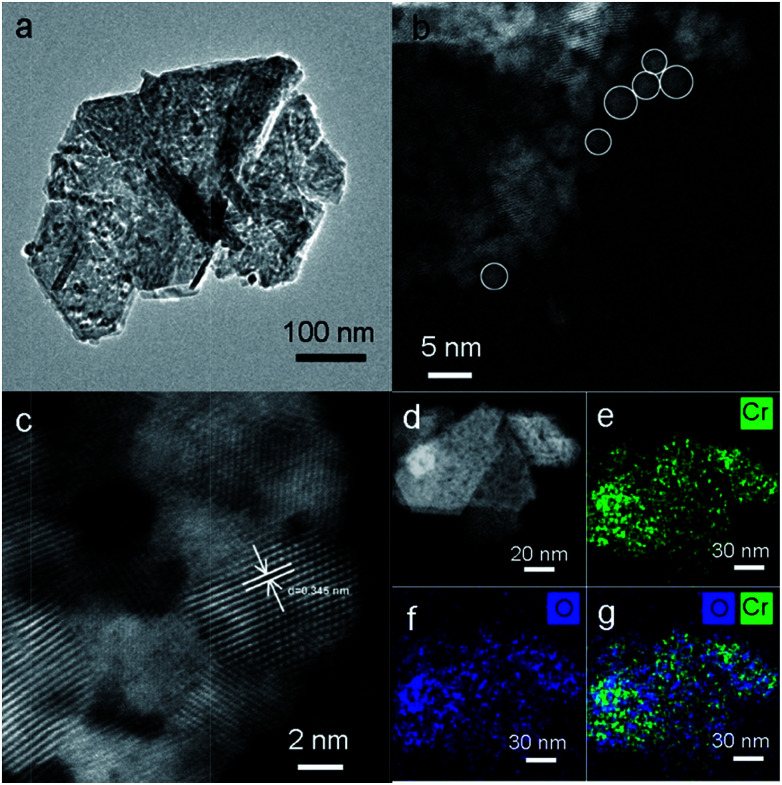
(a) TEM image, (b–d) high resolution HAADF-STEM image of CrO_*x*_/C-450 catalyst. (e, f) 2D STEM EDS elemental maps of Cr-Kα (Green) and O (Blue) elements, respectively. (g) A superimposed image of (e) and (f).

TEM analysis was performed to investigate the structure of MIL-101, CrO_*x*_/C-450, and Cr_2_O_3_/C-WI. As shown in Fig. S5a,† the MIL-101 with octahedron morphology can be observed distinctly, while it transformed to a hexagonal sheet after calcination (Fig. 3a). Further structural information was obtained *via* high-angle annual dark field (HAADF) imaging (Fig. 3b). The hexagonal-like sample observed in Fig. 3a was actually an aggregation of nano-particles with an average size of 3 nm. In addition, lattice fringes of *d* = 0.345 nm were observed (Fig. 3c), corresponding to the eskolaite Cr_2_O_3_ (012) crystal plane, which further confirmed that the Cr oxide in the CrO_*x*_/C-450 catalyst has the eskolaite phase. From the EDX element mapping analysis (Fig. 3e–g), Cr element and O element were observed to be highly dispersed in the CrO_*x*_/C-450 sample and the superimposed image of Cr and O matches the HAADF image. It was estimated that the CrO_*x*_/C-450 catalyst was primarily formed by CrO_*x*_ nano-particles with eskolaite phase as designed in this study. The CrO_*x*_/C-450 sample after 3 poisoning–regeneration cycles was also imaged to investigate the stability of the catalyst (Fig. S5b†). The used sample was primarily composed of CrO_*x*_ nano-particles, similar to the fresh sample, and no major difference in the particle size of Cr_2_O_3_ could be observed after 5 deactivation–regeneration circles, which evidently proved the stability of the sample CrO_*x*_/C-450. Cr_2_O_3_ and Cr_2_O_3_/C-WI, however, had a bulk-like shape (Fig. S5c and d†) with average particle sizes of over 100 nm. In the structure of the MIL-101 precursor, metal nodes containing 3 Cr atoms were covered by organic linkers. Therefore, it is reasonable to suggest that CrO_*x*_ nano-particles in the CrO_*x*_/C-450 catalyst stabilized by covering carbon species transformed from organic linkers of MIL-101 after the calcining process.

**Fig. 4:**
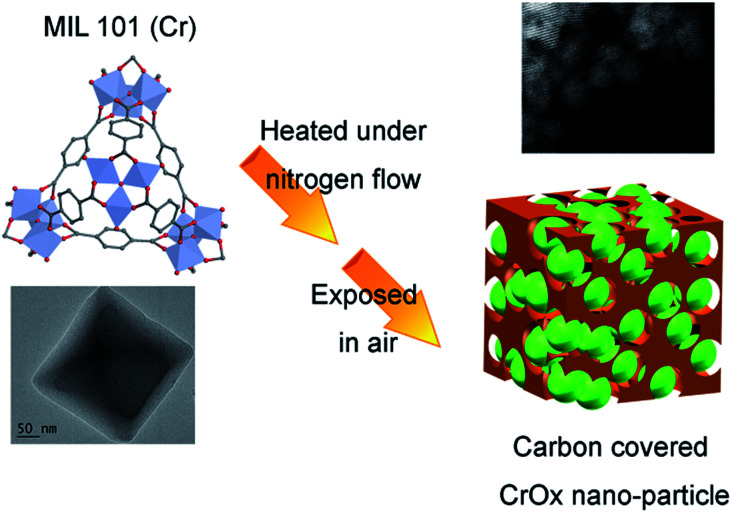
Possible mechanism of catalyst synthesis (green spheres present CrO_*x*_ nano-particles, brown skolen present amorphous carbon).

The feasible mechanism of catalyst synthesis is displayed in Fig. 4. During the calcination process, organic linkers covering Cr nodes carbonized and the structure of MIL-101 gradually destroyed. The amorphous carbon from organic linkers limited the growth of Cr nodes. Finally, when the calcined sample was exposed to air, the remaining Cr nano-particles were oxidized to CrO_*x*_ nano-particles with eskolaite phase, forming the structure of amorphous carbon covered CrO_*x*_ nano-particles (CrO_*x*_/C).

### 3.3. Textural properties of catalysts

As mentioned before, the CrO_*x*_/C-450 catalyst was primarily formed by CrO_*x*_ nano-particles with eskolaite phase and exhibited enhanced NH_3_-SCR activity and satisfying regeneration ability. To explore the relationship between the structure of CrO_*x*_/C-450 and the NH_3_-SCR performance, the properties of the catalysts were characterized by BET, XPS, H_2_-TPR, O_2_-TPD, and NH_3_-TPD analyses.

The surface areas of the catalysts can influence the number of active sites on the catalyst surface, which is considered as an important fact affecting the catalytic activity of NH_3_-SCR catalysts. The surface areas of CrO_*x*_/C-450, Cr_2_O_3_, and Cr_2_O_3_/C-WI obtained from BET analysis are listed in Table 1. The BET surface areas of CrO_*x*_/C-450 and Cr_2_O_3_/C-WI are similar and larger than that of Cr_2_O_3_. Due to the enhanced NH_3_-SCR performances of CrO_*x*_/C-450 and Cr_2_O_3_/C-WI (Fig. 1), it is believed that the enlarged surface area increased the activity of the Cr oxide catalyst. According to the reaction rates normalized by the surface areas of the active material of the catalysts, CrO_*x*_/C-450 exhibited comparatively superior NO conversion than Cr_2_O_3_/C-WI at 125 °C and 150 °C. This infers that a large surface area is not the only reason for the excellent NH_3_-SCR activity of CrO_*x*_/C-450.

The acidity of the catalysts, which can influence the absorption of reaction agents, is an important factor affecting the NH_3_-SCR performance of catalysts. This property of catalysts was investigated by NH_3_-TPD analysis. As displayed in Fig. S6a,† the NH_3_ desorption behaviours of Cr_2_O_3_ and CrO_*x*_/C-450 were similar. No distinct NH_3_ desorption peak was observed from 150 to 400 °C in the profiles of both Cr_2_O_3_ and CrO_*x*_/C-450 samples, which indicated the weak acidity of these two samples. Therefore, the giant NH_3_-SCR performance difference between Cr_2_O_3_ and CrO_*x*_/C is not the result of acidity.

**Fig. 5:**
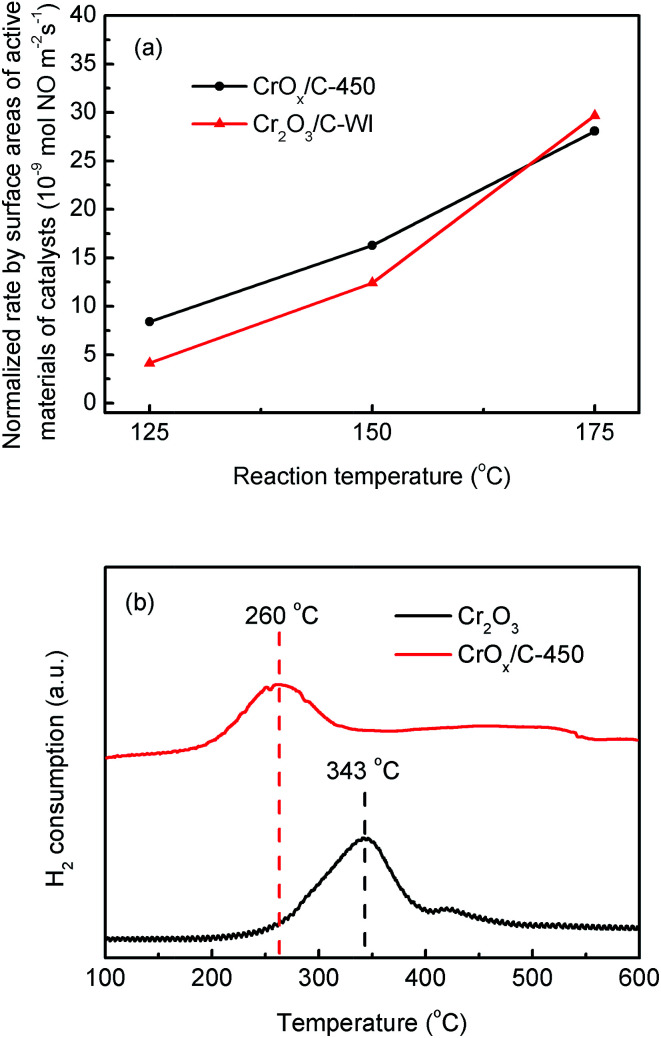
(a) Normalized rates per surface areas of active material of CrO_*x*_/C-450 and Cr_2_O_3_/C-WI catalysts, (b) H_2_-TPR result of CrO_*x*_/C-450 and bulk Cr_2_O_3_.

The redox ability of materials is another significant factor influencing the catalytic activity of the NH_3_-SCR catalyst. H_2_-TPR method was utilized to discuss this property of the synthesized catalysts. As illustrated in Fig. 5b, Cr_2_O_3_ exhibited a single-peak profile. The H_2_ consumption peak at 343 °C was a result of one-step reduction from Cr^6+^ to Cr^3+^, accompanied by the loss of lattice oxygen atoms connected with Cr^6+^ ions.^46^ For the CrO_*x*_/C-450 sample, the H_2_ consumption peak shifted to a low temperature, which indicated that CrO_*x*_/C-450 exhibited stronger oxidation ability and a higher amount of active lattice oxygen than Cr_2_O_3_. In addition, O_2_-TPD analysis was carried out to investigate the stability of the oxygen atoms of the catalyst; the results are shown in Fig. S6b.† In the profile of Cr_2_O_3_, no O_2_ desorption peak was discovered in the temperature range from 50 °C to 450 °C, which indicates that the oxygen on the surface of Cr_2_O_3_ is stable and inert. However, the curve of CrO_*x*_/C-450 exhibited an O_2_ desorption peak from 200 to 350 °C, which was much higher than the desorption temperature of absorbed O_2_ species recorded earlier.^47^ Hence, the desorption peak from 200 to 350 °C was believed to correspond to the dissociation of lattice oxygen from CrO_*x*_/C-450. This demonstrates that the lattice oxygen of CrO_*x*_/C becomes more active and unstable than that of Cr_2_O_3_. Remarkably, the CrO_*x*_/C catalyst has high surface atom/lattice atom rate for its ultrasmall size as mentioned before. It is reasonable to conclude that the unsaturated surface atoms of CrO_*x*_/C cause the unique redox ability exhibited in H_2_-TPR analysis and the instability of the lattice oxygen atom detected in O_2_-TPD analysis. Unstable and activated oxygen atoms are inferred to enhance the activity of CrO_*x*_/C catalyst.

**Fig. 6:**
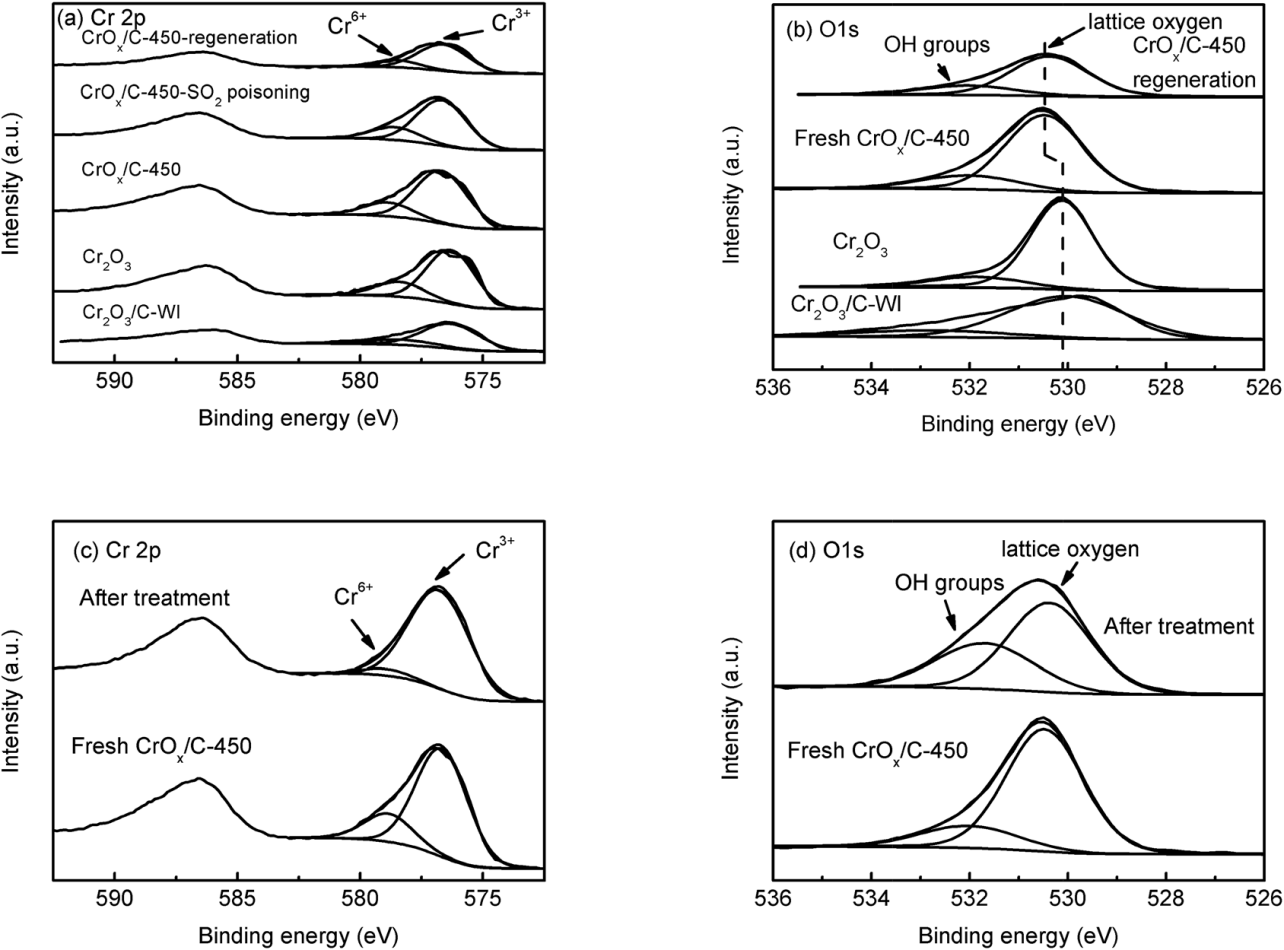
XPS spectra of Cr_2_O_3_, fresh CrO_*x*_/C-450 catalyst, SO_2_ poisoned CrO_*x*_/C-450 catalyst, and regenerated CrO_*x*_/C-450 catalyst: (a) Cr 2p spectra, (b) O 1s spectra, and *ex situ* XPS analysis of fresh CrO_*x*_/C-450 catalyst and CrO_*x*_/C-450 catalyst heated under NH_3_ + NO flow at 150 °C for 1 h: (c) Cr 2p spectra, (d) O 1s.

In XPS analysis, Cr 2p spectra (Fig. 6a) of all the samples were comparable. The Cr 2p_3/2_ peak could be divided into two peaks at 576.7 eV and 578.6 eV, belonging to Cr^3+^ and Cr^6+^, respectively.^48^ The relative contents of Cr^3+^ and Cr^6+^ ions were analogous (Table 2), indicating their similar Cr state. The O 1s peak (Fig. 6b) could be separated into two peaks at 530.1 eV and 531.9 eV, attributed to the lattice oxygen and surface –OH groups, respectively.^49,50^ The relative contents of these two types of oxygen species are also listed in Table 2. The CrO_*x*_/C-450 sample exhibited more surface –OH groups than Cr_2_O_3_. Notably, the peak belonging to the lattice oxygen of CrO_*x*_/C-450 shifts to the high binding energy side, contrasting with that of Cr_2_O_3_ and Cr_2_O_3_/C-WI. It is evident that the lattice oxygen of CrO_*x*_/C-450 carries less negative charge than Cr_2_O_3_ and Cr_2_O_3_/C-WI. Materials with an ultrasmall size are deemed to have abundant dangling bands and their surface atoms are usually unsaturated-coordinated. In case of CrO_*x*_/C-450, some surface oxygen atoms are inferred to be unsaturated coordinated for ultrasmall size of CrO_*x*_ nano-particles. This unsaturated oxygen is considered to have less negative charge and is expected to be more active than the saturated coordinated oxygen of bulk Cr_2_O_3_.

**Table 2:** Relative contents of Cr and O species on surfaces of catalysts

Sample	Cr 2p		O 1s	
	Cr^3+^^a^ (%)	Cr^6+^^a^ (%)	OH groups^a^ (%)	Lattice oxygen^a^ (%)
Cr_2_O_3_	76.74	23.26	13.39	86.61
CrO_*x*_/C-450	78.86	21.14	18.36	81.64
CrO_*x*_/C-450-regeneration	79.37	20.63	18.7	81.3
CrO_*x*_/C-450 *ex situ* treatment	94.87	5.13	36.76	63.24
CrO_*x*_/C-450-SO_2_ poisoning	77.06	22.94	—	—

a Relative contents of Cr and O species were calculated from peak areas ratio of divided peaks in XPS spectra.

Furthermore, to determine whether the lattice oxygen participates in the NH_3_-SCR reaction, *ex situ* XPS analysis was carried out. The CrO_*x*_/C-450 catalyst was heated under NH_3_ + NO flow at 150 °C for 1 h; the XPS spectra of CrO_*x*_/C-450 before and after treatment are displayed in Fig. 6c and d. In the Cr 2p spectra (Fig. 6c), the peak of Cr^6+^ at 578.6 eV disappeared after NH_3_ + NO treatment, while the peak of Cr^3+^ at 576.7 eV enhanced. This infers that Cr^6+^ species on the surface of CrO_*x*_/C can react with the reagent molecules and eventually get consumed. Moreover, in the O 1s spectra (Fig. 6d), the peak intensity of lattice oxygen at 530.4 eV decreased after treatment, while the intensity of the peak attributed to the surface –OH group at 531.7 eV increased, which indicates the loss of surface lattice oxygen. Therefore, lattice oxygen was believed to take part in the NH_3_-SCR on the CrO_*x*_/C catalyst. Thus, CrO_*x*_/C-450, which has more active lattice oxygen, can exhibit enhanced NH_3_-SCR activity than Cr_2_O_3_.

### 3.4. Mechanism of NH_3_-SCR on CrO_*x*_/C catalyst

**Fig. 7:**
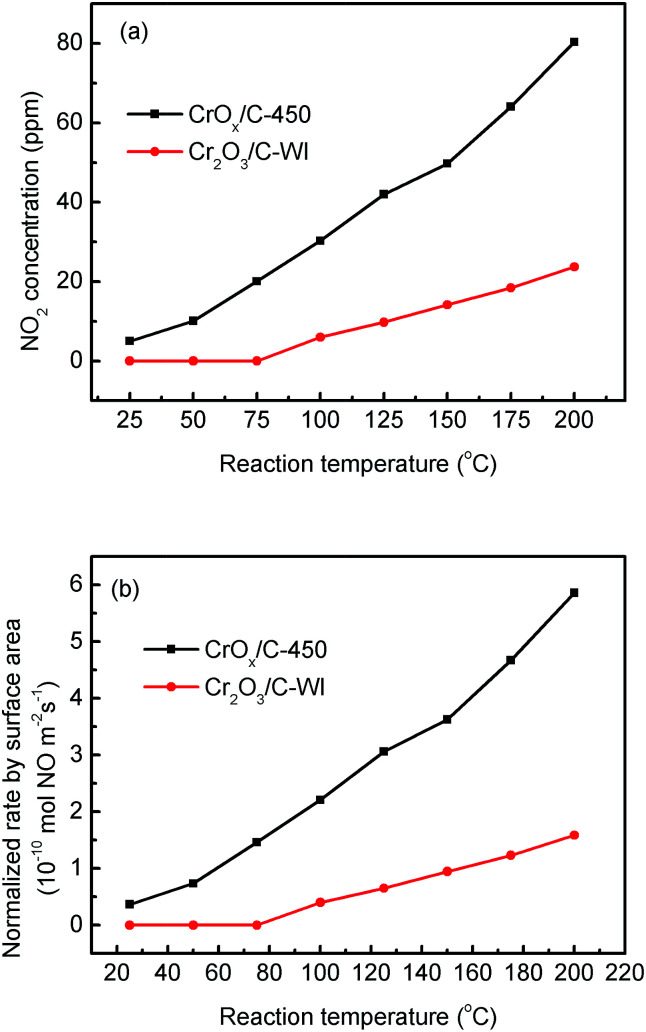
(a) NO oxidation performances and (b) normalized NO oxidation rate by surface area on CrO_*x*_/C-450 and C–Cr_2_O_3_ catalysts.

In “fast NH_3_-SCR”, NO catalytically reduced NH_3_ in assistance of NO_2_, which was reported to have lower activation energy and enhanced catalytic activity compared with the typical NH_3_-SCR. Herein, the CrO_*x*_/C catalyst was proved to have activated lattice oxygen. It is reasonable to deduce that NO can be oxidized to NO_2_ by the activated oxygen on the CrO_*x*_/C surface, making the NH_3_-SCR on CrO_*x*_/C proceed as the “fast NH_3_-SCR” pathway. To verify this conjecture, NO oxidation on CrO_*x*_/C-450 and Cr_2_O_3_/C-WI catalysts was carried out. As displayed in Fig. 7a, NO could be oxidized to NO_2_ on both CrO_*x*_/C-450 and Cr_2_O_3_/C-WI. However, the mass of formed NO_2_ on CrO_*x*_/C-450 exceeded as compared to that on Cr_2_O_3_/C-WI. Moreover, the normalized rate by surface area of NO oxidation on CrO_*x*_/C-450 clearly surpassed that on Cr_2_O_3_/C-WI (Fig. 7b). This indicated that NO is more easily oxidized to NO_2_ on CrO_*x*_/C-450 catalyst with activated oxygen, which probably resulted in the superb NH_3_-SCR performance of CrO_*x*_/C-450.

**Fig. 8:**
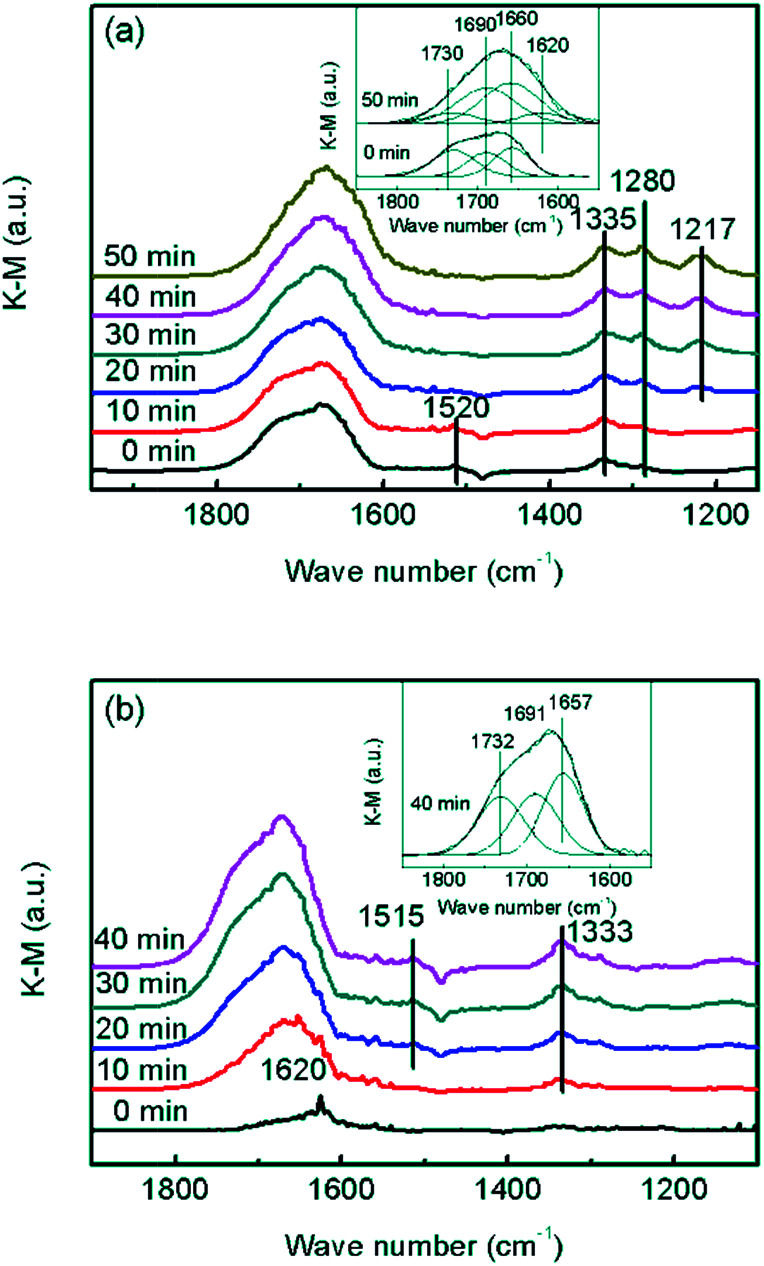
*In situ* DRIFT spectra of CrO_*x*_/C-450: (a) NH_3_ reacted with pre-absorbed NO + O_2_, (b) NO + O_2_ reacted with pre-absorbed NH_3_.

In order to confirm whether the formed NO_2_ participated in NH_3_-SCR on the CrO_*x*_/C catalyst, further information about the reaction mechanism was obtained using DRIFTS. In the spectra of the absorption NO + O_2_ saturated CrO_*x*_/C-450 sample (Fig. 8a), the bands centered at 1280, 1335, and 1520 cm^−1^, and a wide band divided into bands at 1730, 1690, and 1660 cm^−1^ were detected. As reported elsewhere,^51^ these IR bands were attributed to weakly bound NO_2_ (1730 cm^−1^), nitrite anion (1335 cm^−1^), *ν*_s_(N=O) and *ν*_as_(N=O) of symmetric N_2_O_3_ (1690 and 1660 cm^−1^), bidentate nitrates (1520 cm^−1^), and monodentate nitrates (1280 cm^−1^). When the feed gas was switched to NH_3_, the bands belonging to NO_2_ and bidentate nitrates gradually disappeared, replacing with the bands of NH_3_ absorbed on Lewis acid sites (1620 and 1217 cm^−1^),^52,53^ while the bands corresponding to symmetric N_2_O_3_, nitrite anion, and monodentate nitrates were still present. Indeed, it is apparent that NO_2_ and bidentate nitrates participated in the surface reaction on CrO_*x*_/C-450 and were consumed by NH_3_, which is a typical “fast NH_3_-SCR” pathway.^54^ To determine the role of NH_3_, co-absorption of NO + O_2_ on CrO_*x*_/C-450 after pre-absorption of NH_3_ was investigated (Fig. 8b). In the spectra of the absorption NH_3_ saturated CrO_*x*_/C-450 sample, only a weak band at 1620 cm^−1^ corresponding to NH_3_ on Lewis acid sites was detected, indicating the low acidity of CrO_*x*_/C-450 as mentioned in NH_3_-TPD analysis. With the addition of NO and O_2_, this band gradually disappeared, accompanied by the appearance of bands belonging to weakly bound NO_2_ (1732 cm^−1^), nitrite anion (1333 cm^−1^), *ν*_s_(N=O) and *ν*_as_(N=O) of symmetric N_2_O_3_ (1691 and 1657 cm^−1^), and bidentate nitrates (1515 cm^−1^).^51^ This indicated that NH_3_ on Lewis sites was reacted and consumed. Therefore, the entire NH_3_-SCR on CrO_*x*_/C-450 followed a L–H mechanism, in which NO was oxidized by the unsaturated oxygen of CrO_*x*_/C-450 catalyst and transformed to NO_2_. The formed NO_2_ further absorbed on the surface of CrO_*x*_/C-450 as bidentate nitrates, and reacted with NH_3_ on Lewis sites, forming N_2_ and H_2_O at last. Due to the formation of NO_2_, the reaction proceeded as a “fast NH_3_-SCR” pathway, causing the decrease in reaction activation energy, thus enhancing the low-temperature activity.

### 3.5. Reasons of excellent SO_2_ tolerance of regeneration ability

Sulfation process is a transition from metal oxide to metal sulfate. Irrespective of which type of intermediate exists in the sulfation process, the metal–oxygen band must be broken. It is reasonable to deduce that a metal oxide with a strong metal–oxygen band is difficult to be sulfated. The Cr–O band energy of the eskolaite phase Cr_2_O_3_ was calculated (see the ESI†). As displayed in Table S5,† the band energy of the Cr–O band in the eskolaite phase Cr_2_O_3_ exceeds that of Mn–O band in MnO_2_, indicating that the sulfation process of Cr_2_O_3_ proceeds with more difficulty as compare that of MnO_2_. This is considered to be the result of the various NH_3_-SCR performances of MnO_2_ and CrO_*x*_/C-450 catalyst in the presence of SO_2_. Moreover, MnO_2_ and CrO_*x*_/C-450 catalysts deactivated by SO_2_ were washed with deionized water and the metal-ion contents in the eluate were measured by ICP analysis. As presented in Table S6,† Mn^2+^ was detected in the eluate of the deactivated MnO_2_ sample, while no Cr^3+^ was detected in the eluate of the deactivated CrO_*x*_/C-450 sample. It is evident that the CrO_*x*_/C-450 sample was protected from sulfation.

To further understand the SO_2_ poisoning and regeneration processes, the XPS spectra of the fresh CrO_*x*_/C-450 catalyst, SO_2_-poisoned sample, and regenerated sample were studied. As displayed in Fig. 6a and Table 2, the Cr state and relative content of Cr^6+^ and Cr^3+^ of each sample were similar, which indicated that, as it is designed, CrO_*x*_/C-450 catalyst was difficult to be sulfated for the high lattice energy of Cr_2_O_3_. Comparing the O 1s peak of the fresh CrO_*x*_/C-450 sample and the regenerated sample (Fig. 6b), their peaks of lattice oxygen were similar and both shifted to the high binding energy side than that of bulk Cr_2_O_3_. Since activated lattice oxygen still remained on the surface, the regenerated catalyst exhibited high NH_3_-SCR activity, similar to the fresh catalyst.

In XPS spectra of the SO_2_ poisoned sample, the peaks of S 2p and N 1s were detected. The S 2p peak (Fig. S7a†) consists of two peaks at 168.5 eV (S 2p_1/2_) and 169.7 eV (S 2p_3/2_), which belonged to SO_4_^2−^. The N 1s spectra (Fig. S7b†) can be divided into two peaks at 399.5 eV and 400.5 eV, contributed to NH_3_ and NH_4_^+^, respectively. According to the relative atom contents listed in Table S7,† atoms-ratio of SO_4_^2−^ and NH_4_^+^ was nearly 1 : 1 on the surface of the SO_2_-poisoned sample. This indicated that NH_4_HSO_4_ deposited on the surface of CrO_*x*_/C-450 during the SO_2_ poisoning process, which causes the deactivation of the catalyst. After heat treatment, the deposited NH_4_HSO_4_ could easily decompose and the CrO_*x*_/C-450 catalyst with exposed activated lattice oxygen regains the superior activity.

## 4. Conclusions

In this study, we successfully designed and synthesized a novel chromium oxide nano-particles catalyst with excellent NH_3_-SCR activity at 150 °C and remarkable SO_2_ regenerative ability. The obtained CrO_*x*_/C-450 catalyst was composed of CrO_*x*_ nano-particles covered by amorphous carbon. A carbon species, which was derived from the organic linkers of the MOFs precursor, protected the CrO_*x*_ nano-particles from aggregation. CrO_*x*_/C catalysts primarily have Eskolaite phase Cr_2_O_3_ with average size of 3 nm and exhibit a large surface area. Due to the small size of CrO_*x*_ nano-particles in CrO_*x*_/C catalysts, the lattice oxygen atoms of CrO_*x*_/C were activated, so that NO could be oxidized to NO_2_ on the catalyst surface. The formed NO_2_ participated in reaction and made NH_3_-SCR on CrO_*x*_/C proceed through a “fast NH_3_-SCR” pathway. The large surface area and activated lattice oxygen of CrO_*x*_/C catalysts caused the enhanced NH_3_-SCR activities. Due to the stable lattice of Cr_2_O_3_, CrO_*x*_/C catalyst could hardly be sulfated in the SO_2_ poisoning process. Therefore, the regenerated catalyst still retained prominent activity when NH_4_HSO_4_ deposited on the surface of the catalyst was removed during the regeneration process.

## Conflicts of interest

There are no conflicts to declare.

## Supplementary Material

RA-008-C7RA09680A-s001
