# Supplementary material for: Synthesis of CrOx/C catalysts for low temperature NH3-SCR with enhanced regeneration ability in the presence of SO2
Source: RSC Adv. 2018 Jan 22;8(7):3858–68. doi: 10.1039/c7ra09680a (PMC9077754; doi:10.1039/c7ra09680a)
Supplement: RA-008-C7RA09680A-s001 [file RA-008-C7RA09680A-s001.pdf]

Electronic Supplementary Material (ESI) for RSC Advances.
This journal is © The Royal Society of Chemistry 2017

Supporting information

Synthesis of CrO_x/C catalyst for low temperature NH_3-SCR with enhanced regeneration ability in the presence of SO_2

Shuohan Yu[a,b], Sheng Xu[c], Bowen Sun[a,b], Yiyang Lu[a,b], Lulu Li[a,b], Weixin Zou[a,b], Peng Wang[c], Fei Gao[a,b], Changjin Tang[a,b], Lin Dong*[a,b]*

[a.] Key Laboratory of Mesoscopic Chemistry of MOE, School of Chemistry and Chemical Engineering, Nanjing University, Nanjing 210093, PR China

[b.] Jiangsu Key Laboratory of Vehicle Emissions Control, Center of Modern Analysis, Nanjing University, Nanjing 210093, PR China

[c.] National Laboratory of Solid State Microstructures, College of Engineering and Applied Sciences and Collaborative Innovation Center of Advanced Microstructures, Nanjing University, Nanjing 210093, China

Band energy calculation of metal-oxygen band in Cr_2O_3 and MnO_2

Due to the ligancy of Cr in Cr_2O_3 is 6, each Cr_2O_3 unit have 12 Cr-O bands, and thus the enthalpy of Eq. 1 equals 12 times of Cr-O band energy.

$$Cr_2O_3 \rightarrow 2Cr_{(g)} + 3O_{(g)} \qquad (1)$$

Eq. 1 can be depicted as 2×Eq. 3+3/2×Eq. 4-Eq. 2.

$$2Cr_{(s)} + 3/2O_{2(g)} \rightarrow Cr_2O_3 \qquad (2)$$

$$Cr_{(s)} \rightarrow Cr_{(g)} \qquad (3)$$

$$1/2O_{2(g)} \rightarrow O_{(g)} \qquad (4)$$

Enthalpies of Eq. 2-4 are formation enthalpy of Cr_2O_3 $\Delta H_f(Cr_2O_3)$, vaporization enthalpy of Cr solid $\Delta H_{vap}(Cr)$, and formation enthalpy of oxygen atom $\Delta H_f(O)$, respectively. Therefore, band energy of Cr-O band E_{Cr-O} can be expressed as Eq. 5.

$$E_{Cr-O} = \frac{2\times\Delta H_{vap}(Cr) + 3\times\Delta H_f(O) - \Delta H_f(Cr_2O_3)}{12} \qquad (5)$$

Analogously, band energy of Mn-O band in MnO_2 E_{Mn-O} can be expressed as Eq. 6.

$$E_{Mn-O} = \frac{\Delta H_{vap}(Mn) + 2\times\Delta H_f(O) - \Delta H_f(MnO_2)}{6} \qquad (6)$$

Table S1. Thermochemical data of some metal oxide.

Metal oxide	Sulfating process	Gibbs free energy (kJ/mol)[a]	Melting point (^oC)
Cr_2O_3	$2Cr_2O_3 + 6SO_2 + 3O_2 \rightarrow 2Cr_2(SO_4)_3$	-1139.54	2435
CoO	$2CoO + 2SO_2 + O_2 \rightarrow 2CoSO_4$	-463.265	1933
Fe_2O_3	$2Fe_2O_3 + 6SO_2 + 3O_2 \rightarrow 2Fe_2(SO_4)_3$	-1070.86	1539
CuO	$2CuO + 2SO_2 + O_2 \rightarrow 2CuSO_4$	-409.611	1326
MnO_2	$MnO_2 + SO_2 \rightarrow MnSO_4$	-172.871	535

[a] Thermodynamic data was obtained from Lange's Chemistry Handbook Version 13th.

Table S2. Kinetics data of NH_3-SCR on CrO_x/C-450 and Cr_2O_3/C-WI catalysts.

Samples	CrO_x/C-450			Cr_2O_3/C-WI		
Reaction temperature (^oC)	125	150	175	125	150	175
$[NO]_{out}$	453	409	343	489	467	421
NO conversity (%)	9.32	18.1	31.2	2.15	6.46	15.7

Table S3. Kinetics data of NH_3-SCR on CrO_x/C-450 and Cr_2O_3/C-WI catalysts, when NH_3 concentration was maintained at 500 ppm.

Reaction temperature (°C)	125		150		175	
Feed gas content (ppm)	$[NO]_{in}$	$[NO]_{out}$	$[NO]_{in}$	$[NO]_{out}$	$[NO]_{in}$	$[NO]_{out}$
	205	183	205	167	205	151
CrO_x/C-450	146	133	146	120	146	100
	88	78	88	71	88	62
	128	125	128	119	128	108
Cr_2O_3/C-WI	63	60	63	55	63	41
	215	212	215	207	215	194

Table S4. Kinetics data of NH_3-SCR on CrO_x/C-450 and Cr_2O_3/C-WI catalysts, when NO concentration was maintained at 500 ppm.

Reaction temperature (°C)	125		150		175	
Feed gas content (ppm)	$[NO]_{in}$	$[NO]_{out}$	$[NO]_{in}$	$[NO]_{out}$	$[NO]_{in}$	$[NO]_{out}$
	178	162	178	145	178	122
CrO_x/C-450	111	100	111	86	111	66
	55	47	55	38	55	26
	140	133	140	120	140	97
Cr_2O_3/C-WI	60	56	60	45	60	31
	212	204	212	187	212	160

Table S5. Band energy calculation of metal-oxygen band in Cr_2O_3 and MnO_2.

Metal oxide	Cr_2O_3	MnO_2
Formation enthalpy of metal oxide (kJ/mol)[a]	-1134.97	-520.15
Vaporization enthalpy of metal solid (kJ/mol)[a]	397.58	280.81
Formation enthalpy of oxygen atom (kJ/mol)[a]	249.23	
Band energy of metal-oxygen band (kJ/mol)	223.15	216.57

[a] Thermodynamic data was obtained from Lange's Chemistry Handbook Version 13th.

Table S6. Mass of metal sulfate formed on catalysts.

catalysts	Metal sulfate	$m_{M^{n+}}$ (mg/L)	m_{MSO_4} (mg)
MnO_2	$MnSO_4$	34.4	4.7
CrO_x/C-450	$Cr_2(SO_4)_3$	0	0

Table S7. Surface atomic concentration of SO_2 poisoned CrO_x/C-450 sample.

S_{total} (%)	N_{total} (%)	NH_3 (%)	NH_4^+ (%)
2.69	3.63	1.08	2.55

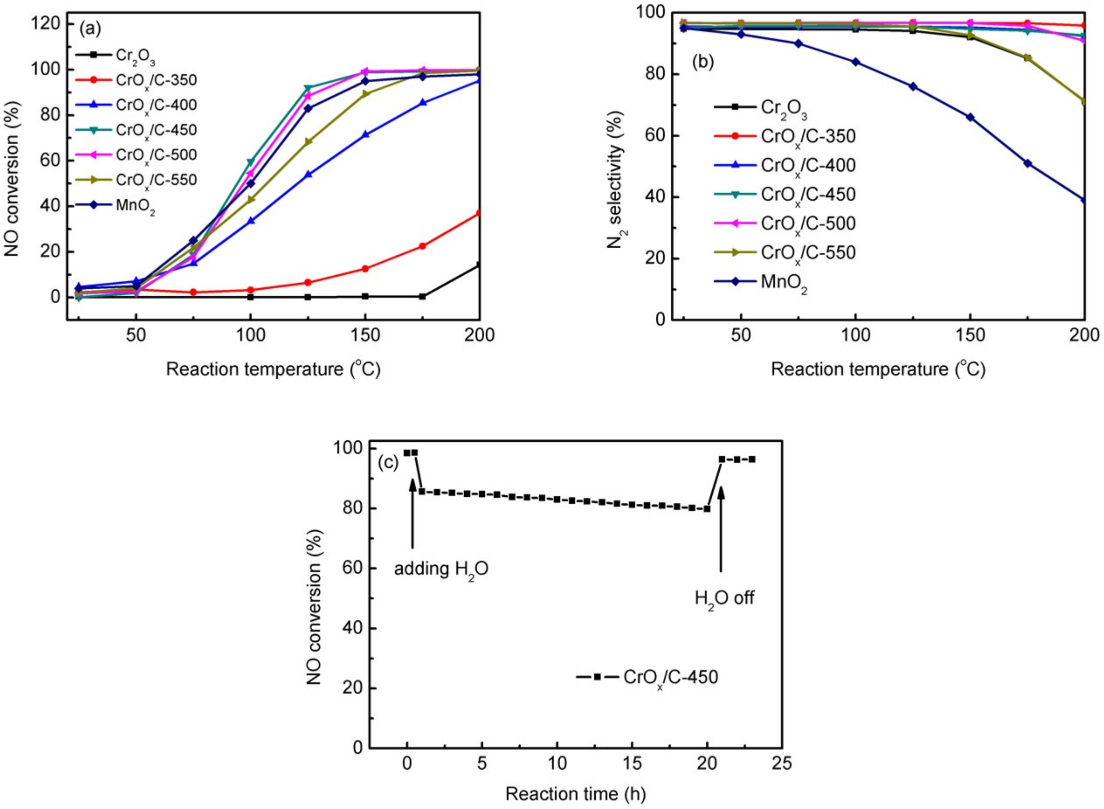

Figure S1. NH_3-SCR activity of catalysts derived from MIL-101 and Cr_2O_3: (a) NO conversion, (b) N_2 selectivity, and (c) water tolerance of CrO_x/C-450.

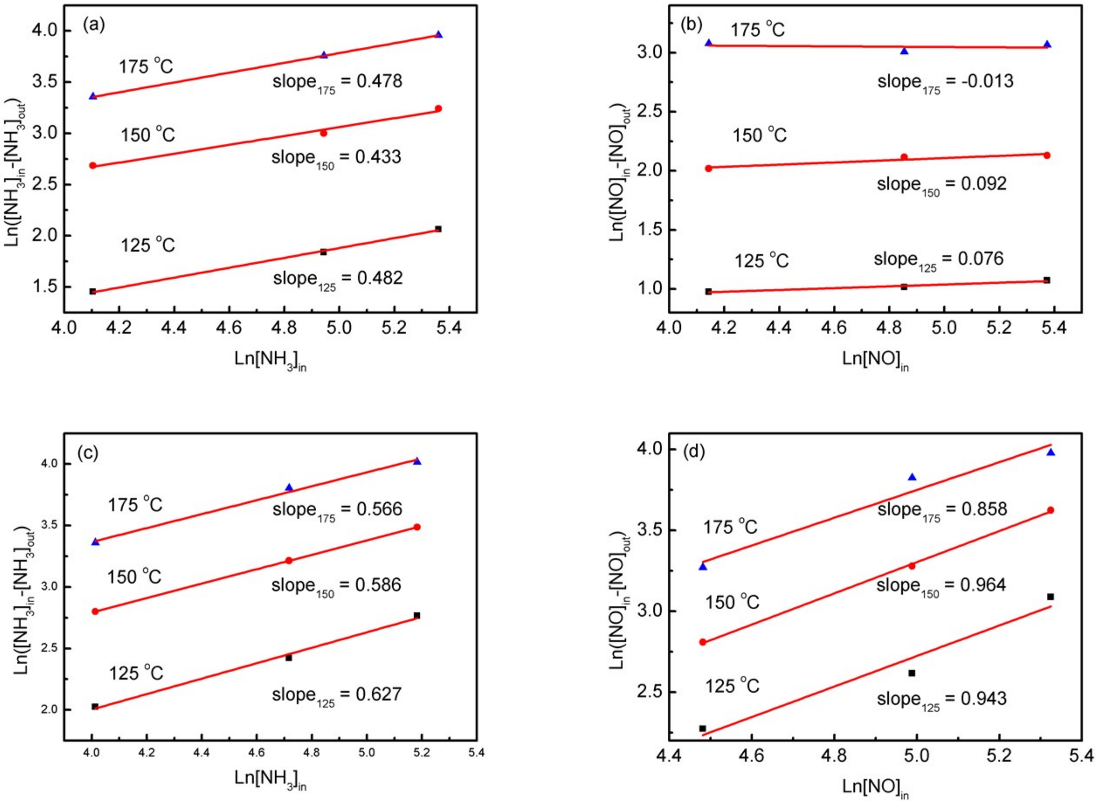

Figure S2. (a, b) Kinetics curves of NH_3-SCR on Cr_2O_3/C-WI catalysts: (a) NO concentration was maintained at 500 ppm, (b) NH_3 concentration was maintained at 500 ppm, and (c, d) Kinetics curves of NH_3-SCR on CrO_x/C-450 (a) NO concentration was maintained at 500 ppm, (b) NH_3 concentration was maintained at 500 ppm.

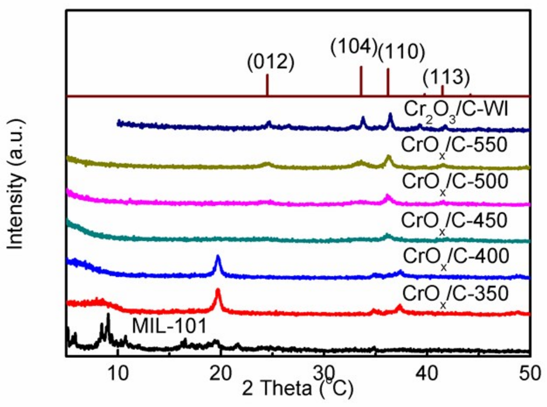

Figure S3. XRD patterns of catalysts.

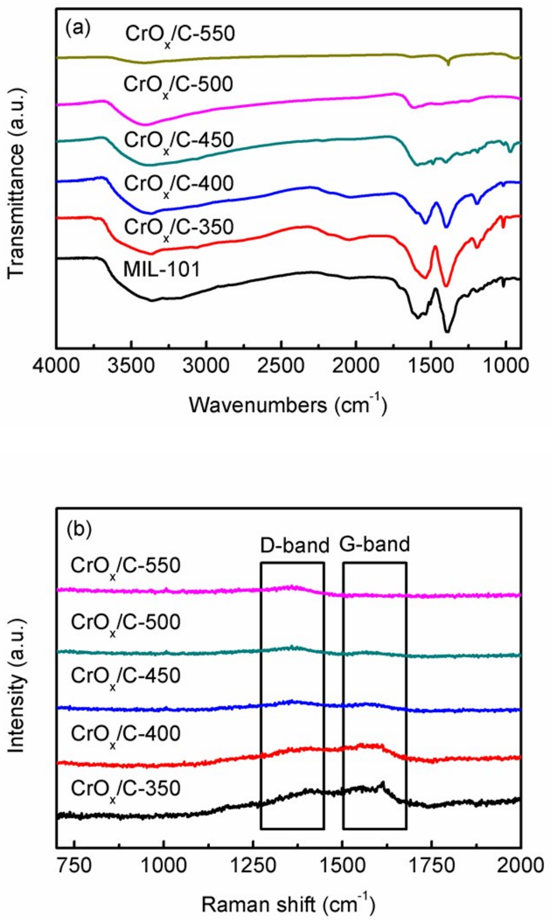

Figure S4. (a) FTIR and (b) Raman profiles of catalysts and MIL-101.

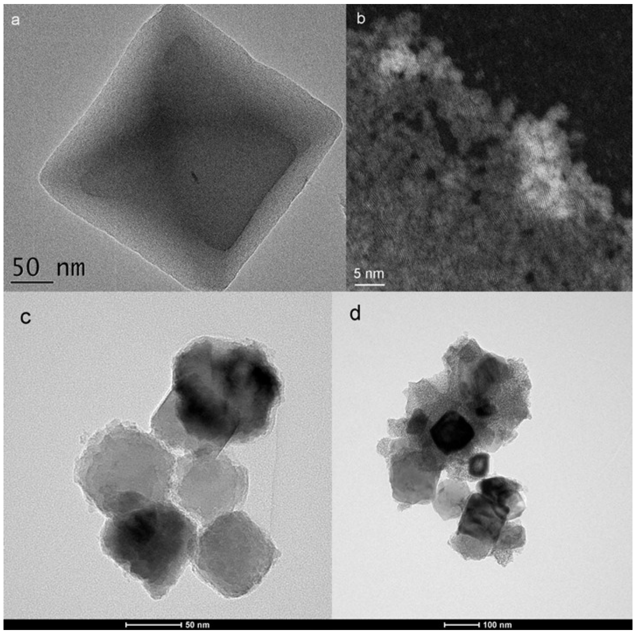

Figure S5. (a) TEM image of MIL-101, (b) HAADF image of regenerated CrO_x/C-450 catalyst, (c) TEM image of bulk Cr_2O_3, and (d) TEM image of Cr_2O_3/C-WI.

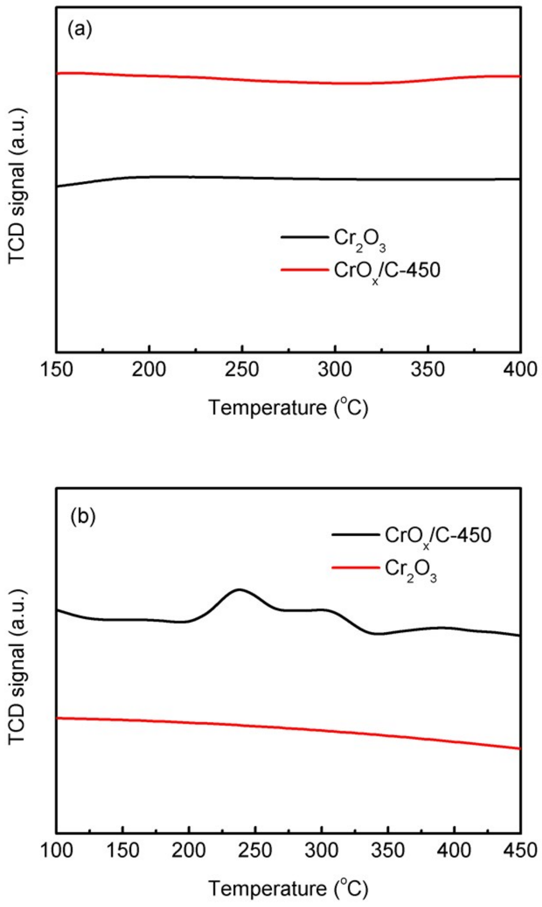

Figure S6. (a) NH_3-TPD, and (b) O_2-TPD profiles of CrO_x/C-450 and Cr_2O_3.

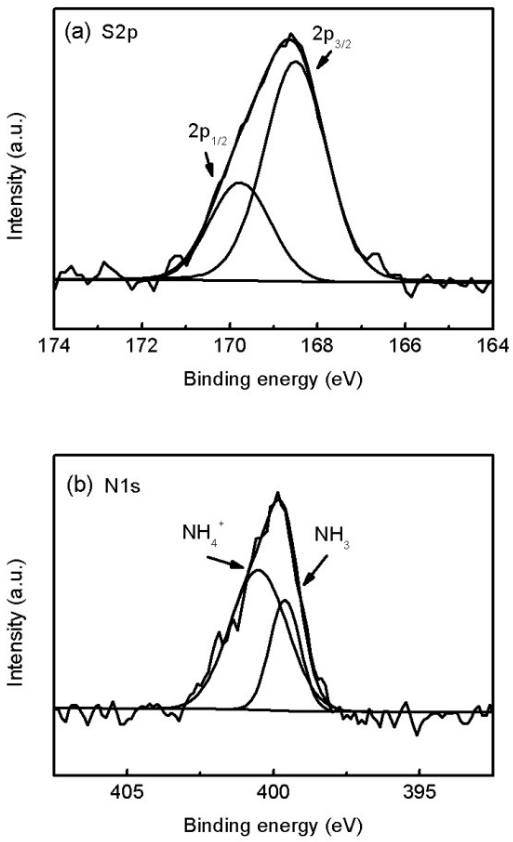

Figure S7. XPS spectra of SO_2 poisoned CrO_x/C-450 catalyst: (a) S2p, and (b) N1s.
